# Cardiac Surgery in Jehovah's Witness Patients: Experience of a Brazilian Tertiary Hospital

**DOI:** 10.21470/1678-9741-2017-0012

**Published:** 2017

**Authors:** Felipe Homem Valle, Fernando Pivatto Júnior, Bruna Sessim Gomes, Tanara Martins de Freitas, Vanessa Giaretta, Miguel Gus

**Affiliations:** 1 Hospital de Clínicas de Porto Alegre (HCPA), Cardiology Division, Porto Alegre, RS, Brazil.; 2 Hospital de Clínicas de Porto Alegre (HCPA), Internal Medicine Division, Porto Alegre, RS, Brazil.

**Keywords:** Jehovah's Witnesses, Cardiac Surgical Procedures, Mortality

## Abstract

**Introduction:**

The outcomes of Jehovah's Witness (JW) patients submitted to open heart surgery may vary across countries and communities. The aim of this study was to describe the morbidity and mortality of JW patients undergoing cardiac surgery in a tertiary hospital center in Southern Brazil.

**Methods:**

A case-control study was conducted including all JW patients submitted to cardiac surgery from 2008 to 2016. Three consecutive surgical non-JW controls were matched to each selected JW patient. The preoperative risk of death was estimated through the mean EuroSCORE II.

**Results:**

We studied 16 JW patients with a mean age of 60.6±12.1 years. The non-JW group included 48 patients with a mean age of 63.3±11.1 years (*P*=0.416). Isolated coronary artery bypass graft surgery was the most frequent surgery performed in both groups. Median EuroSCORE II was 1.29 (IQR: 0.66-3.08) and 1.43 (IQR: 0.72-2.63), respectively (*P*=0.988). The mortality tended to be higher in JW patients (18.8% *vs.* 4.2%, *P*=0.095), and there was a higher difference between the predicted and observed mortality in JW patients compared with controls (4.1 and 18.8% *vs.* 2.1 and 4.2%). More JW patients needed hemodialysis in the postoperative period (20.0 *vs.* 2.1%, *P*=0.039).

**Conclusion:**

We showed a high rate of in-hospital mortality in JW patients submitted to cardiac surgery. The EuroSCORE II may underestimate the surgical risk in these patients.

**Table:** 

Abbreviations, acronyms & symbols	
JW	= Jehovah's Witness
HCPA	= Hospital de Clínicas de Porto Alegre
SPSS	= Statistical Package for Social Sciences
VAP	= Ventilator-associated pneumonia

## INTRODUCTION

Based on religious beliefs, Jehovah's Witness (JW) refuse blood products transfusions. In some clinical situations, it may be both, a healthcare and an ethical challenge. Despite the development in surgical techniques, more than 50% of patients receive perioperative transfusion in cardiac surgeries^[1]^.

More recently, the data of case series^[2-6]^ and some controlled studies^[7-10]^ showed that the perioperative and postoperative prognosis of JW is similar to those of patients who do not have restrictions to blood products transfusions. However, the rates of mortality and postoperative complications in patients that undergo cardiac surgery are variable. Although clinical results are determined largely by sample characteristics and by the preoperative and postoperative care, assistant teams cultural and religious factors may play a specific role in the surgical success of these patients. Therefore, the evaluation of the cardiac surgery results in JW patients should be evaluated in different cultural scenarios.

In Brazil, there are no studies that address local results in cardiovascular procedures in such context. The 2010 Brazilian census^[11]^ showed that 1,393,208 persons (0.73% of whole population) were identified as JW. The aim of this study was to describe the morbidity and mortality of JW patients undergoing cardiac surgery in a tertiary hospital center of Porto Alegre, Southern Brazil, considering only the more contemporary cases. We also compare the predicted mortality estimated by the EuroSCORE II^[12]^ in JW patients and controls.

## METHODS

The current case-control study was carried out at the Hospital de Clínicas de Porto Alegre (HCPA), a tertiary hospital in Southern Brazil (state of Rio Grande do Sul), during the period from 2008 to 2016. All JW patients submitted to cardiac surgery were selected. The patients' identification as JW occurred through surgical schedules, bioethics consultations and keyword search in the electronic medical records system. Three consecutive surgical non-JW controls were matched to each selected JW patient, including only surgeries with extracorporeal circulation.

Preoperative risk of death was estimated through the mean EuroSCORE II^[12]^. Death during hospitalization, regardless of its length, was defined as hospital mortality. The registry of at least one of the following complications was considered as hospital morbidity: creatinine > 2 mg/dL, mechanical ventilation > 48 hours, myocardial infarction, need for either hemodialysis or intra-aortic balloon pump, reintervention due to bleeding, reintubation, stroke and use of antibiotics. Definitions of active endocarditis, chronic pulmonary disease, critical preoperative state, surgery urgency, extracardiac arteriopathy and recent myocardial infarction (< 90 days) were the used in the EuroSCORE II study^[12]^. Creatinine clearance was estimated through Cockroft-Gault formula.

Data were collected directly from the patients' electronic charts, and analyzed in the software Statistical Package for Social Sciences (SPSS) 21.0. Qualitative data were reported as absolute and relative frequency; mean (± standard deviation) or median (interquartile range) were used for quantitative variables. The comparison of the groups was performed by Student's t-test for quantitative variables with normal distribution, by Mann-Whitney U test, for the quantitative without normal distribution and chi-square test for categorical variables. In situations of low frequency, Fisher exact test was used. Normality of the distribution of each variable was evaluated using Shapiro-Wilk test. The significance level adopted in all tests was 5%. The present study was submitted and approved by the local Research Ethics Committee.

## RESULTS

During the period under study, 16 JW patients were submitted to cardiac surgery at the institution. The demographic characteristics of the whole sample are described in Table 1. Patients were neither receiving iron supplementation therapy nor were in critical state in the preoperative period.

**Table 1:** Demographic characteristics of the sample.

Variable	JW (n=16)	Non-JW (n=48)	*P*
Age	60.6±12.1	63.3±11.1	0.416
Male sex	9 (56.3)	32 (66.7)	0.652
Systemic hypertension	14 (87.5)	41 (85.4)	1.0
Previous smoking	6 (37.5)	29 (60.4)	0.192
Current smoking (< 30 days)	__	5 (10.4)	0.319
Chronic pulmonary disease	__	1 (2.1)	1.0
Pulmonary hypertension (≥ 31 mmHg)	5 (31.2)	13 (27.1)	0.756
Previous MI	5 (31.3)	15 (31.3)	1.0
Recent MI	1 (6.3)	10 (20.8)	0.265
Diabetes	4 (25.0)	17 (35.4)	0.645
Diabetes on insulin	__	4 (8.3)	0.564
NYHA class III/IV heart failure	3 (18.7)	9 (18.8)	1.0
LVEF	56.0 (37.5-67.0)	55.5 (42.2-68.7)	0.951
CCS class 4 angina	1 (6.3)	8 (16.7)	0.430
Unstable angina	__	1 (2.1)	1.0
Previous heart surgery	1 (6.3)	2 (4.2)	1.0
Atrial fibrillation	1 (6.3)	6 (12.5)	0.669
Extracardiac arteriopathy	1 (6.3)	6 (12.5)	0.669
Active endocarditis	1 (6.3)	__	0.250
Creatinine clearance (mL/min)*	88.6 (50.4-102.3)	72.2 (55.1-98.9)	0.617
Preoperative hemodialysis	1 (6.3)	__	0.250
Acetylsalicylic acid use	6 (37.5)	28 (58.3)	0.247
Erythropoietin use*	1 (6.7)	__	0.238
Hematocrit (%)	39.7 (35.2-42.6)	37.4 (33.1-41.7)	0.438
Hemoglobin (g/dL)	13.6 (11.7-14.2)	12.7 (11.1-14.2)	0.571
EuroSCORE II	1.29 (0.66-3.08)	1.43 (0.72-2.63)	0.988

CCS=Canadian Cardiovascular Society; JW=Jehovah's Witness; LVEF=left ventricular ejection fraction; MI=myocardial infarction; NYHA=New York Heart Association

* Excluding a chronic kidney disease on hemodialysis patient.

Data presented as number (%), mean ± standard deviation or median (interquartile range).

Isolated coronary artery bypass graft surgery was the most frequent surgery performed in both groups. Extracorporeal circulation and cross-clamp times were similar between JW and non-JW groups. Surgical characteristics data are described in Table 2.

**Table 2:** Surgical data.

Variable	JW (n=16)	Non-JW (n=48)	*P*
Non-elective surgery	1 (6.3)	4 (8.3)	1.0
Surgery			
Isolated CABG	7 (43.8)	28 (58.3)	0.469
Isolated biological AVR	3 (18.8)	6 (12.5)	
Isolated biological MVR	2 (12.5)	1 (2.1)	
CABG + biological AVR	1 (6.3)	1 (2.1)	
CABG + biological aortic valved graft	1 (6.3)	__	
Isolated mechanical AVR	1 (6.3)	3 (6.3)	
Mechanical AVR + MVR	1 (6.3)	__	
Isolated mechanical MVR	__	2 (4.2)	
Mechanical aortic valved graft + aneurysmectomy	__	1 (2.1)	
CABG + aorta pseudoaneurysm correction	__	1 (2.1)	
Mechanical aortic valved graft	__	1 (2.1)	
Biological aortic valved graft	__	1 (2.1)	
Heart tumor removal	__	1 (2.1)	
Interventricular communication correction	__	1 (2.1)	
Resection of subaortic membrane + septoplasty	__	1 (2.1)	
Extracorporeal circulation time (minutes)	58.5 (50.7-71.5)	67.5 (55.2-90.0)	0.139
Cross-clamp time (minutes)	38.5 (31.2-51.0)	48.0 (40.0-65.0)	0.054

AVR=aortic valve replacement; CABG=coronary artery bypass graft surgery; JW=Jehovah's Witness; MVR=mitral valve replacement Data presented as number (%) or median (interquartile range).

Hospital outcomes are presented in Table 3. There was no statistically significant difference in the rate of mortality or morbidity, with a trend to a higher mortality in the JW group. Causes of death were septic (n=1), cardiogenic (n=1) and hypovolemic (n=1) shock in the JW group; ischemic stroke (n=1) and right ventricle failure/shock (n=1) were responsible for the deaths in the control group. The levels of both hematocrit and hemoglobin at discharge were leveled between the two groups. Lengths of stay, considering both intensive care unit and ward stay after surgery, were also similar between groups.

**Table 3:** Hospital outcomes.

Outcome	JW (n=16)	Non-JW (n=48)	*P*
Mortality	3 (18.8)	2 (4.2)	0.095
Morbidity	4 (25.0)	14 (29.2)	1.0
Last hematocrit (%)	28.6 (23.6-33.6)	28.8 (26.5-32.9)	0.625
Last hemoglobin (g/dL)	9.2 (7.5-11.6)	9.4 (8.8-10.9)	0.593
Length of stay (days)	6.5 (6.0-9.5)	7.0 (7.0-9.7)	0.143

JW=Jehovah's Witness. Data presented as number (%) or median (interquartile range).

**Fig. 1:**
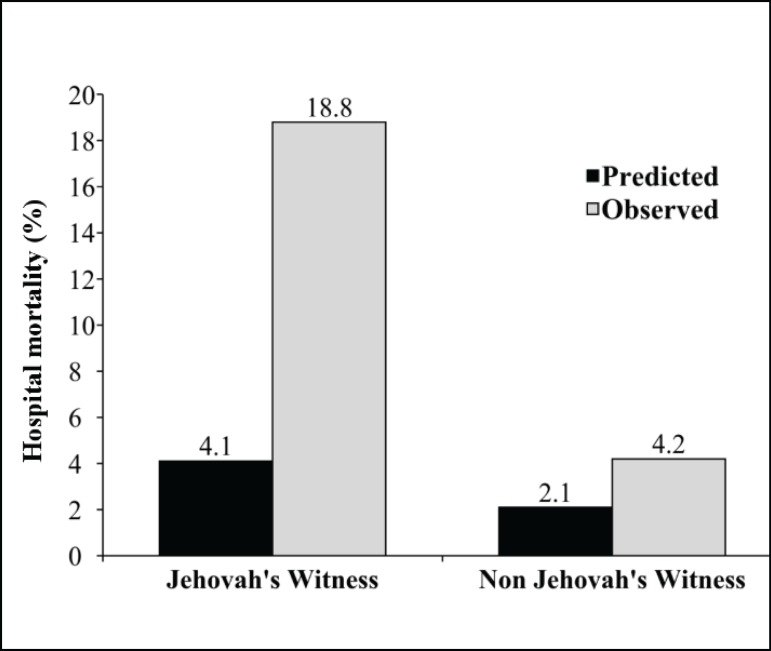
Comparison of the predicted and observed hospital mortality in JW and non-JW patients accordingly EuroSCORE II.

The comparison of the predicted and observed mortality is shown in Figure 1. As noted, unlike non-JW group, the observed mortality was higher than the rate predicted by mean EuroSCORE II in the JW group.

The need for hemodialysis in the postoperative period was significantly higher in JW patients, but the incidence of the other morbidities analyzed was similar between the patients' groups. Detailed hospital morbidity per outcome is shown in Table 4. The reasons for antibiotic use were septic shock due to central line infection (n=1) and ventilator-associated pneumonia (VAP; n=1) in JW patients; respiratory tract infection (n=4), urinary tract infection (n=2), surgical wound infection (n=2), VAP (n=1) and diverticulitis (n=1) accounted for the use of antibiotics in the non-JW group.

**Table 4:** Hospital morbidity.

Hospital morbidity	JW (n=16)	Non-JW (n=48)	*P*
Mechanical ventilation > 48h	4 (25.0)	4 (8.3)	0.099
Need for hemodialysis*	3 (20.0)	1 (2.1)	0.039
Reintubation	3 (18.8)	3 (6.3)	0.159
Antibiotic use^†^	2 (13.3)	10 (20.8)	0.714
Creatinine > 2 mg/dL*	1 (6.7)	4 (8.3)	1.0
Perioperative MI	1 (6.3)	2 (4.2)	1.0
Need for IABP	1 (6.3)	__	0.250
Reintervention for bleeding	1 (6.3)	3 (6.3)	1.0
Stroke	__	2 (4.2)	1.0

JW=Jehovah's Witness; IABP=intra-aortic balloon pump; MI=myocardial infarction

* Excluding a chronic kidney disease on hemodialysis patient.

† Excluding an active endocarditis patient.

Data presented as number (%).

## DISCUSSION

In this case-control study, we reported the hospital outcomes of a non-selected group of JW that were submitted to cardiac surgery in a Brazilian tertiary center between 2008 and 2016. Outcomes and demographic variables were compared with a matched control group as described above. The rates of hospital mortality and morbidity were leveled between JW and controls. However, there was a trend toward higher mortality rate in JW than in controls (18.8 *vs.* 4.2%, respectively; *P*=0.095). In addition, necessity of hemodialysis in the postoperative period was greater in JW than in controls (20.0 *vs.* 2.1%, respectively; *P*=0.039). Moreover, it was observed that, in JW, the mortality rates were higher than predicted by the EuroSCORE II. Hemoglobin levels remained similar between groups, both preoperatively and at discharge.

Previous retrospective studies demonstrated that cardiac surgery might be performed in JW with acceptable outcomes^[2-10]^. Furthermore, retrospective studies that compared mortality and morbidity rates in JW and controls showed leveled results between both groups^[7-10]^. Bhaskar et al.^[9]^ and Pattakos et al.^[10]^ compared outcomes of JW with a control group of transfused patients. Marinakis et al.^[7]^ and Stamou et al.^[8]^ described outcomes of JW with a matched group regardless of blood transfusion. Table 5 shows a comparison of current study with previous retrospective comparative studies. In agreement with previous comparative studies, we observed similar levels of hemoglobin between groups, both preoperatively and at discharge. Our results also showed similar rate of reoperation due to excessive bleeding in JW and in controls. However, the in-hospital mortality rate in JW in our study was higher than in previous studies. In addition, our report is the first to demonstrate both higher necessity of hemodialysis in the postoperative period and a trend toward higher mortality rate in JW than in controls. Notwithstanding, our cohort is the first report that demonstrates higher mortality rates in JW than predicted by the EuroSCORE II in all risk strata.

**Table 5:** Comparison of current study with previous retrospective comparative studies.

Variable	Valle et al. (current study)	Marinakis et al.^[7]^	Stamou et al.^[8]^	Bhaskar et al.^[9]^	Pattakos et al.^[10]^
n	16	31	49	49	322
Age	60.6±12.1	62±15	62.7±9.5	65.3±10.1	62±13
Isolated CABG	7 (43.8)	15 (48.4)	38 (77.5)	25 (51.0)	209 (64.9)
Hospital mortality	3 (18.8)	1 (3.2)	3 (6.1)	1 (2.0)	10 (3.1)

CABG=coronary artery bypass graft surgery Data presented as number (%) or mean ± standard deviation.

Our study has several limitations. First, our sample of JW was small. However, this is a non-selected and consecutive cohort of JW and there is no record of denial of cardiac surgery to any JW at our hospital. Second, surgical data were heterogeneous between our groups: the rates of combined surgery and valve surgery were higher in JW than in controls. This can partly explain a trend toward higher mortality rate among JW in our cohort. Third, this is a cross-sectional retrospective study with all methodological limitations of such design. Therefore, our results need to be interpreted in a cautious and exploratory fashion.

## CONCLUSION

In conclusion, our study demonstrated a high rate of in-hospital mortality in JW and a trend toward higher mortality in JW than in controls. In addition, we observed that in our cohort of JW the mortality risk predicted by EuroSCORE II was not accurate: in fact, EuroSCORE II underestimated surgical risk in JW in our study. To our knowledge, this is the first Brazilian study to compare outcomes of heart surgery in JW with controls.

**Table:** 

Authors' roles & responsibilities	
FHV	Substantial contributions to the conception or design of the work; or the acquisition, analysis, or interpretation of data for the work; drafting the work or revising it critically for important intellectual content; final approval of the version to be published
FPJ	Substantial contributions to the conception or design of the work; or the acquisition, analysis, or interpretation of data for the work; drafting the work or revising it critically for important intellectual content; final approval of the version to be published
BSG	Substantial contributions to the conception or design of the work; or the acquisition, analysis, or interpretation of data for the work; final approval of the version to be published
TMF	Substantial contributions to the conception or design of the work; or the acquisition, analysis, or interpretation of data for the work; final approval of the version to be published
VG	Substantial contributions to the conception or design of the work; or the acquisition, analysis, or interpretation of data for the work; final approval of the version to be published
MG	Substantial contributions to the conception or design of the work; or the acquisition, analysis, or interpretation of data for the work; drafting the work or revising it critically for important intellectual content; final approval of the version to be published
